# Severe laryngeal edema caused by *Pseudoterranova* species: A case report

**DOI:** 10.1097/MD.0000000000024456

**Published:** 2021-01-29

**Authors:** Shiori Suzuki, Nobuyuki Bandoh, Takashi Goto, Akihiro Uemura, Mizuki Sasaki, Yasuaki Harabuchi

**Affiliations:** a Department of Otolaryngology-Head and Neck Surgery, Hokuto Hospital, Inadacho Kisen 7-5, Obihiro; b Department of Otolaryngology-Head and Neck Surgery; c Department of Parasitology, Asahikawa Medical University, Midorigaoka-Higashi 2-1-1-1, Asahikawa, Hokkaido, Japan.

**Keywords:** allergic reaction, *Anisakiasis*, base of tongue, laryngeal edema, *Pseudoterranova*

## Abstract

**Rationale:**

Severe laryngeal edema can cause upper airway obstruction, which is fatal. *Pseudoterranova*, an uncommon nematode of the family Anisakidae, predominantly invades the stomach after ingestion of the nematodes in raw or undercooked marine fish. There have been a few reports of development of severe laryngeal edema caused by the nematode invading the base of the tongue.

**Patient concerns:**

A 69-year-old Japanese woman complained of stuffy and scratchy throat for 8 hours and reported eating sashimi, fresh slices of raw jacopever, 4 days before the first visit.

**Diagnosis:**

Endoscopy revealed a white-yellowish wriggling worm at the left side of the base of the tongue and severe edema of the larynx.

**Interventions:**

The worm was extracted using endoscopic forceps. The patient was hospitalized and treated with intravenous injection of an antibiotic and steroid.

**Outcomes:**

The symptoms and laryngeal edema disappeared the next day. The worm was identified as a 4th-stage larva of *Pseudoterranova* spp based on morphologic features. The serum *Anisakis*-specific IgE antibody level was high, at 38.6 UA/mL.

**Lessons:**

Clinicians should be aware of the possibility of severe laryngeal edema due to invasion by anisakid nematodes in the pharyngolaryngeal area in cases involving previous ingestion of raw or uncooked marine fish.

## 1 Introduction

Laryngeal edema is caused by several conditions, including a viral or bacterial infection known as acute epiglottitis,^[1,2]^ allergic reactions such as angioedema or anaphylaxis in association with ingesting of foods or drugs,^[3]^ and trauma of the larynx. Severe laryngeal edema sometimes causes upper airway obstruction, which is fatal.

Anisakiasis, an emerging foodborne zoonosis, is caused by infection with members of the genera *Anisakis* or *Pseudoterranova* in the Anisakidae family.^[4]^*Anisakis* spp., a major type member of the family, is a white nematode that causes gastric, intestinal, and ectopic anisakiasis, as well as allergic diseases.^[5]^ Similar to *Anisakis* spp., *Pseudoterranova* spp. predominantly invades the stomach after ingestion of 3rd-stage larvae contained in raw or undercooked marine fish. Approximately 20,000 anisakiasis cases are reported annually worldwide, and over 90% of cases are reported in Japan, followed by Spain.^[4,5]^

There have been a few reports of invasion by anisakid nematodes in the oral and pharyngolaryngeal area. Furthermore, little is known about how invasion by *Pseudoterranova* causes severe laryngeal edema. We herein report a rare case of severe laryngeal edema occurring 4 days after the patient ingested raw marine fish.

## 2 Case report

**Figure 1:**
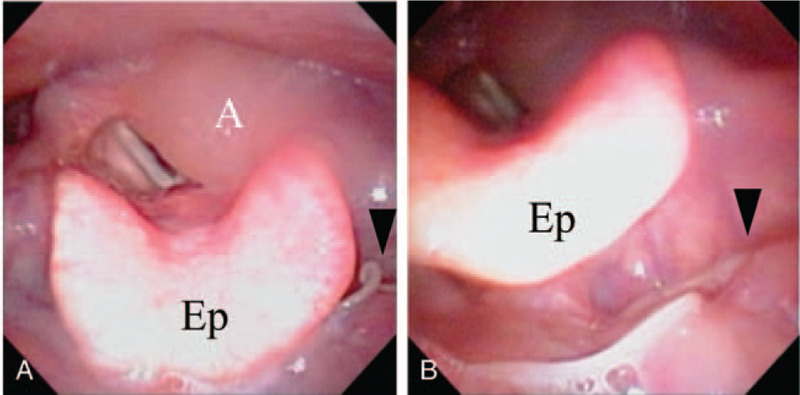
Endoscopic examination showed severe edema of the left arytenoid and slight edema of the epiglottis (A). A white-yellowish wriggling worm was observed at the left side of the base of the tongue (B). Site of invasion is shown by arrowhead. A = arytenoid, Ep = epiglottis.

A 69-year-old Japanese woman who complained of stuffy and scratchy throat for 8 hours visited the emergency department. The patient reported that she had ingested sashimi, fresh slices of raw jacopever, 4 days before the first visit and had not ingested any raw marine fish since then. She had no medical history except for hypertension and hyperlipidemia. Endoscopic examination showed severe edema of the left arytenoid and slight edema of the epiglottis (Fig. 1A). The left-side vocal cord and the glottis could not be seen. A white-yellowish wriggling worm was observed at the left side of the base of the tongue (Fig. 1B). We extracted the worm using endoscopic forceps. Laboratory examination showed slightly elevated inflammation factors: peripheral blood white cell count of 9310/μL (normal range: 3500-9700/μL) and C-reactive protein level of 0.54 mg/dL (normal range: < 0.3 mg/dL). The patient was hospitalized and treated with intravenous injection of 3 g of ampicillin/sulbactam twice a day and 250 mg of hydrocortisone succinate once. The symptoms and laryngeal edema disappeared the next day. The patient was discharged 3 days after being admitted. The result of serum total immunoglobulin (Ig)E collected the day after the first visit was elevated at 193 IU/mL (normal range: <170 IU/mL). *Anisakis*-specific IgE antibody level measured by ImmunoCAP-FEIA was high at 38.6 UA/mL (normal range: <0.34 UA/mL) and categorized as class 4. Other serum specific-IgE levels for fishes such as salmon, mackerel, horse mackerel, sardine, and squid were not elevated.

**Figure 2:**
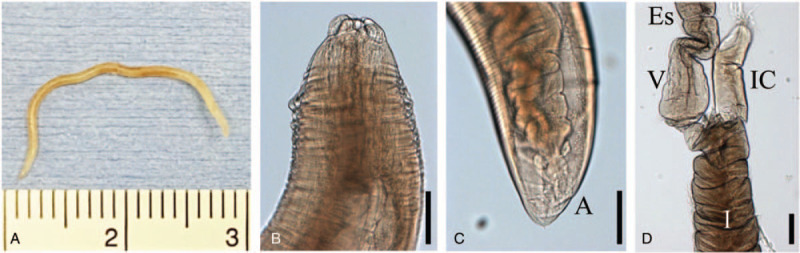
Measurements of the worm were as follows: body 25 mm long and 1.0 mm wide (A). The worn had white-yellowish colored body covered in part by brown color. The anterior end of the worm contained 3 lips and the bilobed medial region of the 3 lips was prominent (B). The tail was conical and had a small process at the posterior end (C). Intestinal cecum extending anteriorly along the ventriculus was observed (D). The worm was identified as a 4th-stage larva of *Pseudoterranova* spp.. A = anus, Es = esophagus, I = intestine, IC = intestinal cecum, V = ventriculus; Scale bar: 200 μm.

The worm was fixed in 10% formalin and cleared in alcohol glycerin. The worm was 25 mm long and 1.0 mm wide and had white-yellowish colored body covered in part by brown color (Fig. 2A). The anterior end of the worm contained 3 lips, and the bilobed medial region of the 3 lips was prominent (Fig. 2B). The tail was conical and had a small process at the posterior end (Fig. 2C). Reproductive organs were not developed. Intestinal cecum extending anteriorly along the ventriculus was observed (Fig. 2d). On the basis of morphologic features, the worm was identified as a 4th-stage larva of *Pseudoterranova* spp..

## 3 Discussion

In the natural reproductive cycles of anisakid nematodes, the adult form of *Anisakis* spp. lives in the intestines of marine mammals such as whales and dolphins, whereas that of *Pseudoterranova* spp. lives in mammals such as seals and sea lions.^[5]^ Their eggs are passed by fecal excretion into the sea water. The eggs become embryonated in water, form 1st-stage larvae, molt to form 2nd-stage larvae, and finally hatch into free-swimming larvae, which are then eaten by intermediate hosts such as small crustaceans, in which they develop into 3rd-stage larvae. Subsequently, the infective crustaceans are ingested by edible fish, which may then be eaten by humans. Infected fish are eaten by marine mammals and then the larvae grow to the 4th-stage and subsequently the adult stage. *Pseudoterranova* spp. can be distinguished from *Anisakis* spp. by the presence of the intestinal cecum and the body is larger and darker than that of *Anisakis* spp.^[5]^ Fourth-stage larvae of *Pseudoterranova* spp. differ from 3rd-stage larvae in that the bilobed medial region of the lips appears much more prominent.^[6]^ It was reported that *Pseudoterranova* larvae developed from the 3rd to 4th stages during the time of infection in humans, as in our case.^[7]^ The main transmitters of *Pseudoterranova* spp. according to a survey in Hokkaido, the northern island of Japan, are reportedly Pacific cod (*Gadus macrocephala*) in 62 (52%), Pacific halibut (*Hippoglossus stenolepis*) in 24 (20%), fat greenling (*Hexagrammos otakii*) in 8 (7%), and jacopever (black rock cod, *Sebastes schlegeli*) in 5 (4%) of 119 patients diagnosed with anisakiasis.^[8]^ In the present case, jacopever was suspected to contain larvae of *Pseudoterranova* spp.. Fresh slices of raw jacopever are often ingested as sashimi in the Hokkaido area.

Anisakiasis in the oral and pharyngolaryngeal area, at least in part ectopic anisakiasis, is extremely rare, and the etiology remains unknown. To date, in the English literature, only 8 cases of anisakiasis with the identification of invasion of the oral and pharyngolaryngeal area have been reported, and these cases are summarized in Table 1.^[9–15]^ The median age of these patients was 38 years, with a large range of 6 to 69 years. In terms of gender ratio, it was similar (female: 5, male: 3). Nematodes were *Anisakis* spp. in 6 and *Pseudoterranova* spp. in 2 patients. Foods ingested included squid in 2 and tuna, cuttlefish, and jacopever in 1 patient each. The initial symptoms included abnormal sensation or pain in the throat. The location of invasion was the tonsil in 3 cases, and the soft palate, larynx, lip, dorsum of the tongue, and base of the tongue in 1 patient each. The base of the tongue, back third of the tongue and a part of the oropharynx, is the area that cannot be seen through the mouth. Oropharyngeal infection by anisakid nematodes is known to cause “tingling throat syndrome,” and anisakid nematodes are often spit out by coughing immediately after ingestion.^[16]^ Immediate onset of symptoms, within 30 minutes of ingestion, occurred in 4 of 7 patients. In the present case, the period from ingestion to onset of symptoms was 4 days, and the longest of 7 patients as shown in Table 1. We suspected that the local allergic reaction in the present case could be related to the delayed appearance of the initial symptoms. The symptoms and/or findings disappeared after removal of the nematode immediately in 4 and the next day in 2 of 7 patients. One patient remained hospitalized for 10 days prior to discharge due to prolonged tonsillar swelling associated with allergic reactions.^[12]^

**Table 1:** Clinical characteristics of reported cases of Anisakiasis in the oral and pharyngolaryngeal area.

No.	Age/ Gender	Nematode	Food	Initial symptom	Location	Period from ingestion to onset of symptom	Period from ingestion to consultation	Period from removal to disapperance of symptom	Author
1	6/F	Anisakis spp.	Mackerel, Prawn, or Sardine	–	Tonsil	–	–	–	Bhargava (1996)^[9]^
2	35/M	Anisakis spp.	Squid	Throat pain	Soft palate	Immediately	2 hours	Immediately	Kumagai (2006)^[10]^
3	41/F	Anisakis spp.	(Raw fish)	Foreign body sensation	Larynx	3 days	5 days	Immediately	Kwak (2012)^[11]^
4	68/M	Anisakis simplex	Tuna	Sore throat	Tonsil	Immediately	3 days	10 days	Takano (2016)^[12]^
5	39/M	Anisakis spp.	Cuttlefish	Oral pain	Lip	30 mins	1 day	1 day	Choi (2017)^[13]^
6	38/F	Anisakis spp.	Squid	Sensation of sticking	Dorsum of tongue	10 hrs	10 hrs	Immediately	Kumagai (2018)^[14]^
7	25/F	Pseudoterranova azarasi	(Sashimi)	Pharyngeal pain	Tonsil	Immediately	5 days	Immediately	Fukui (2020)^[15]^
8	69/F	Pseudoterranova spp.	Jacopever	Staffy and scratchy throat	Base of tongue	4 days	4 days	1 day	Present case

M = male, F = female, –: unknown.

Allergic reactions occur in cases of re-exposure to *Anisakis*-related antigens in food after sensitization by the primary infection with anisakid nematodes. Allergic reactions generally occur within hours after ingestion of contaminated fish. Symptoms of allergic reactions associated with anisakiasis range from urticaria and angioedema to life-threatening anaphylactic shock.^[4]^ Antigens derived from *Pseudoterranova* spp. have allergenic activity and can induce sensitization in a few days.^[17]^*Pseudoterranova* spp. contain allergens similar to *Anisakis* spp., and they are cross-reactive.^[18]^ The hypothesis of the present case is as follows. The patient had not been previously sensitized to *Pseudoterranova* spp.. A *Pseudoterranova* larva invaded to the base of the tongue over the course of 4 days after the patient ingested slices of raw jacopever. The patient did not exhibit either abnormal sensation in the throat or an allergic reaction. Sensitization to *Pseudoterranova* spp. occurred within 4 days. The patient complained of stuffy and scratchy throat 4 days after ingestion due to severe laryngeal edema caused by a local allergic reaction and in part by infection. Sensitization was proven by the elevated IgE titer for *Anisakis* (which has cross-reactivity to *Pseudoterranova* spp.) based on blood samples collected 5 days after ingestion.

To prevent upper airway obstruction, severe laryngeal edema requires a prompt and appropriate response. The patient needed to be hospitalized and treated with intravenous injection of antibiotics and steroids. It is essential that airway management, including intubation or tracheostomy in case of dyspnea, is implemented. Removal of the worm is the most effective approach in anisakiasis. In particular, transnasal endoscopic removal with forceps is useful for a worm at the base of the tongue, which cannot be seen thorough the mouth. For primary prevention, fish can be ingested after either freezing at −20°C for over 24 hour, or heating to 60°C for 1 minute or above 70°C, as indicated by the Ministry of Health, Labor and Welfare of Japan. The US Food and Drug Administration recommends that fish be kept frozen at −20°C or below for 7 days or frozen at −35°C or below for 15 h.^[19]^

In conclusion, in patients with severe laryngeal edema, clinicians should be aware of the possibility of invasion by anisakid nematodes in the pharyngolaryngeal area in cases involving previous ingestion of raw or uncooked marine fish.

## Acknowledgments

We thank Prof. Hideo Hasegawa of the Department of Infectious Disease Control, Oita University Faculty of Medicine, for assistance in morphological analyses.

We thank FORTE Science Communications (www.forte-science.co.jp) for editing a draft of this manuscript.

## Author contributions

**Formal analysis:** Mizuki Sasaki.

**Project administration:** Takashi Goto, Akihiro Uemura.

**Supervision:** Yasuaki Harabuchi.

**Writing – original draft:** Shiori Suzuki.

**Writing – review and editing:** Nobuyuki Bandoh.

## Abbreviations

Abbreviation: Ig = immunoglobulin.
